# The Relationship between Workplace, Job Stress and Nurses’ Tobacco Use: A Review of the Literature

**DOI:** 10.3390/ijerph7052362

**Published:** 2010-05-11

**Authors:** Pantelis Perdikaris, Eleni Kletsiou, Elpida Gymnopoulou, Vasiliki Matziou

**Affiliations:** 1 General Children’s Hospital of Athens «Panagiotis & Aglaia Kyriakou», Thivon & Livadias Str, GR 11523, Athens, Greece; 2 Coronary Care Unit University Hospital of Athens “Attikon”, 1 Rimini Str, GR 12462, Athens, Greece; E-Mail: eklets@nurs.uoa.gr; 3 Faculty of Medicine, National & Kapodestrian University of Athens, Mikras Asias Str, GR 11523, Athens, Greece; E-Mail: egymnopoulou@nurs.uoa.gr; 4 Faculty of Nursing, National & Kapodestrian University of Athens, 123 Papadiamantopoulou Str, GR 11523, Athens, Greece; E-Mail: vmatziou@nurs.uoa.gr

**Keywords:** smoking, nurses, workplace, job stress, tobacco use

## Abstract

The aim of this study was to provide a summary of the existing published knowledge on the possible relationship between the workplace as a stressor factor and nurses’ tobacco use. A systematic review of the literature from 1995 to 2009, using the MEDLINE database took place. Studies, that referred to nurses’ smoking habit exclusively or as a part of the study, were included in the review. 491 studies were retrieved and their titles/abstracts were examined systematically. Twenty one studies were retrieved for further consideration by a comprehensive literature review. Ten studies fulfilled the eligibility criteria and they were examined further. There is a conflict on the possible relationship between workplace as a stressor factor and nurses’ smoking habits, because there is no evidence on if the nurses’ work environment causes smoking initiation.

## 1. Introduction

Smoking has been identified by the World Health Organization as a global epidemic [1,2]. The crucial role of health care professionals in tobacco control can be demonstrated by the fact that the number of smokers would decrease by an additional two million per year, if only 100,000 health care providers were to help just 10% of their patients to stop smoking [3]. Nurses, as well as physicians, have an important role in educating their patients. They are seen as symbols of good health practice and they can influence the smoking habits of their patients by counseling them on smoking cessation interventions [4]. Nurses-led interventions for smoking cessation increase the chance of successful quitting by 50% [5]. However, there are barriers in achieving this goal by the nurses and therefore it is estimated that only 20% to 30% of nurses provide smoking cessation interventions to their patients [5]. The main barrier is nurses’ smoking behavior [6,8–15]. There are three main reasons that may explain why nurses smoke: stress caused by the working environment, peer and social influence and socioeconomic status and education [7]. Nurses who smoke perceive themselves as not being credible role models to help their patients quitting smoking. There are many studies demonstrating that a strong barrier to conducting smoking cessation interventions with the patients is the nurses themselves who continue to smoke and lack of confidence in their ability to support patients in their efforts to stop smoking [6,8–13]. Nurses who smoke seem to be less willing to take part in such practices and they are more likely to hold attitudes that might reduce the effects of their advice [14,15]. Nurses who smoke, may also be less supportive of smoke-free policy at health-care facilities [16].

Several studies mention that smoke-free health care facilities, like other smoke-free workplaces, can have an impact on nurses’ ability to quit [17–19]. Hospitals may be a potential factor that leads nursing staff to smoke in order to cope against stressful situations caused by the nursing environment. There is no clear link between working environment and tobacco use among nurses, as a lot of nurses have started smoking before entering hospitals, even before commencing training [7,20–23]. Older studies that examined nurses’ smoking behavior, had attempted to demonstrate that smoking as a coping mechanism against stress was associated to the nursing environment [24–30]. There were studies that presented the view that most of the nurses were women whose smoking was described to be related to stress at work, dissatisfaction with work and lack of social support [25,31,32]. The lack of controlling their own work, as well as stressful and high expectations increased smoking in women [33]. The reasons that were given by Leathar’s study for tobacco consumption were: not knowing anyone and having to make friends, having to cope with more responsibility than one should for his/her status on night shift and the general lack of support given by trained staff [24]. Although these studies tried to identify that the nursing environment may cause stress, they failed to comprehensively demonstrate a clear link between stress in the workplace and tobacco use [7]. Rowe and Macleod Clark performed a systematic review about the reasons that nurses smoke and they concluded that nurses were subject to the same kinds of stress as other females and thus were smoking for the same reasons [7]. The relationship between job stress and nicotine dependence could be stronger in hospitals with higher smoking prevalence among nurses, because it was speculated that nurses in such hospitals would be more allowed to smoke when feeling stressful during their assignment [34]. The aim of this systematic review is to provide a summary of the existing published knowledge on the nurses’ tobacco consumption as a coping mechanism against stressful working environment.

## 2. Methodology

A systematic review of the existing literature on the link between stress caused by the working environment and nurses’ tobacco use was carried out. The question posed was: “Is there an association between the stressful working environment and the beginning of tobacco use or the increase in smoking rates among nursing staff as a coping mechanism?” A review protocol was drawn up following standards outlined by the MOOSE Guidelines for Meta-Analysis and Systematic Reviews of Observational Studies [35]. The review was undertaken using the computer database of the US National Library of Medicine Medline for the years 1995–2009, with the help of the PubMed interface. Key words that were used for retrieving studies were: “Working Environment” or “Hospital” or “Nursing Environment” and “Smoking” or “Tobacco Use” or “Tobacco Consumption” and “Nurse” or “Nursing Staff” and “job stress”. The keywords were used all together and in pairs in order to retrieve the best possible number of studies. A single search string was not used, but we paired the different keywords in multiple ways. For example by using the keywords “hospital” and “smoking, 370 studies were retrieved. An additional 31 studies were retrieved by using the keywords “hospital” and “tobacco use”. Three studies were retrieved with the use of the keywords “working environment” and “smoking” *etc.* Studies on human subjects, published in English language that referred to nurses’ smoking habit exclusively or as a part of the study, were candidates for inclusion in the review Retrieved studies were checked against a list of eligibility criteria, while references of each study were checked manually in order to find additional studies that may meet the eligibility criteria.

Inclusion criteria were a priori defined in order to include or exclude the studies. These criteria included: (a) reference to human beings, (b) publication in English language, (c) time interval between 1995 and 2009, (d) epidemiological studies (of any design), (e) reference to nurses’ smoking habit exclusively or as a part of the study. The studies were searched without restrictions and then eliminated studies not in English or not with humans. Studies that did not meet these criteria were excluded from the review. Two researchers, who were working separately, used a standardized data extraction form in order to extract data from every included study systematically. The researchers independently reviewed each of the 491 titles and abstracts when they were available. Any disagreement was noted and discussed in a common meeting by the researchers. A major disagreement was the inclusion of two studies referred to nursing aids instead of nurses. In Greece, nursing aids’ duties overlap those of nurses several times. As nursing aids’ tasks may overlap those of nurses several times, it was decided to be included in the review. Moreover, these studies mentioned the smoking habit of nursing aids and the potential effect of stressful working environment, thus they were relative to the aim of the review, although the sample was not consisted of nurses. Data extracted from each study included study main characteristics, study population, sampling method, hospitals or nursing wards, topic, statistics and conclusions.

## 3. Results

**Figure 1.**
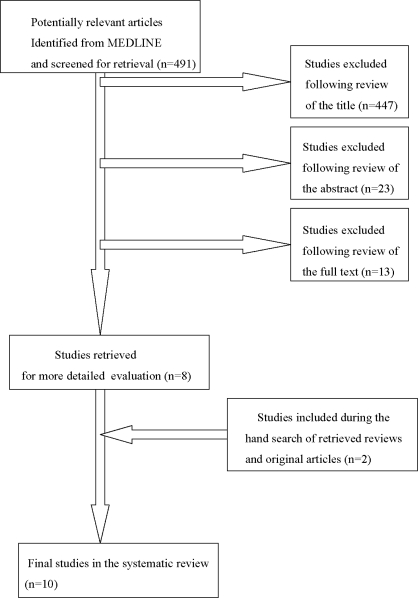
Literature research and strategy outcomes.

491 studies were retrieved by using the above key words. The number of studies identified and selected or excluded in each phase of the research is shown in Figure 1. Additionally, seven studies were provided by manual searching of the bibliography. However, only two of them met the eligibility criteria and were ultimately included in the systematic review. Ten studies were deemed suitable for inclusion in the review, although there were studies (3) where the link between working environment as a stressor factor and nurses’ smoking habits was not so clear.

The main characteristics of the studies included in the systematic review are presented in Table 1. Four prospective cohort studies and four cross-sectional studies were included in the systematic review. Two studies were conducted in the USA, while seven were conducted in Europe (Poland, Northern Ireland, Greece, Norway, and Denmark) and one in Asia (Japan).

Table 2 summarizes the main findings as well as the conclusions of the retrieved studies, regarding the association between the working environment as a stressor factor and the tobacco consumption by the nurses. Job stress at the workplace was described in terms of psychological demand, decision latitude, supervisor support and coworker support [34] or by working more than 18 hours per week [36]. Eriksen’s study mentioned the social climate and the exposure to threats and violence as factors that caused stress at work [37]. There were studies where job stress was described as working at night [38] or having heavy physical job strain [39].

Five studies examined the association between workplace, stress levels and nurses’ smoking behavior or attitudes and experiences. Sarna *et al.* conducted a survey of eight focus groups with current or former smokers (n = 60). Smokers indicated higher levels of stress in the current job (6.9 ± 1.6) in a scale range 1–10 with higher scores indicating greater stress. Former smokers reported lower stress levels than current smokers (5.7 ± 2.7). As Sarna *et al.* mentioned “smoking was described as a way of taking time out and coping with the stressful environment of the busy hospital” [40]. Ota *et al*. performed a cross-sectional study in order to reveal a possible relationship between perceived job demand and self-reported nicotine dependence. They found a correlation between nicotine dependence scores and job stress scales among current smokers (n = 39). In particular, there were four job stress scales: psychological demand, decision latitude, supervisor support and coworker support. Statistically significant correlation was found between nicotine dependence and psychological demand (p < 0.001). The same result arose by multiple regression analysis while controlling for age (standardized beta TDS = 0.417, p = 0.009) [34].

McKenna *et al.* estimated that 68.4% of the nurses who were smokers had increased their consumption once they began their nursing career, while 65.3% of the ex-smokers had increased the tobacco use thereafter. 3.2% of smokers and 44.5% of ex-smokers had taken up the habit after beginning nursing [20]. Another study of McKenna *et al.* quoted nurses’ reasons for smoking. Work pressure was found to be the third reason (5.32 ± 3.23), following addiction (7.13 ± 3.1) and enjoyment (6.56 ± 2.84) in a scale range 1–10 with 1 representing “not important” and 10 being “important” [15]. Tselebis *et al.* examined nursing staff’s anxiety and smoking habits and it was found that smokers’ anxiety scores correlated strongly and positively with their per day quota of cigarettes (Pearson’s r = +0.65, p < 0.002) [41].

Five studies examined the role of work environment and work factors on smoking cessation among nurses. Sanderson *et al.* found that nurses who were working night shift (OR = 0.63, CI: 0.44–0.89) or having physical job strain (OR = 0.51, CI: 0.33–0.80) were less likely to quit smoking [39]. Eriksen conducted two surveys about work factors and smoking cessation in nurses’ aides and work factors as predictors of smoking relapse in the same occupational group [36, 37]. When work factors on smoking cessation were examined, it was found that nurses’ aides who were working 19–36 hours per week (OR = 0.35, CI: 0.13–0.91) or more than 36 hours per week (OR = 0.27, CI: 0.09–0.78) were less likely to quit smoking [36]. When work factors were examined as predictors of smoking relapse, Eriksen found that an increased risk of relapse was associated with the lowest level of the social climate (OR = 2.12, CI: 1.03–4.36), as well as with the reporting of rather often or very often exposure to threats and violence at work during the previous 2 years (OR = 2.08, CI: 1.01–4.29) [37].

Cofta and Staszewski found that hospital staff being on call or working at night were consuming more cigarettes per day than those who were not working at night (17.5 ± 7.7 and 14.4 ± 5.8 respectively). Furthermore, hospital staff on call or working at night were decreasing the time interval of the first cigarette after waking up (<30 min: 58.5% and 31–60 min: 32.1%) in comparison to those who were not working at night (<30 min: 54.7% and 31–60 min: 30.7%), although there was no statistical significance (p = 0.67) [38]. Sarna *et al.* found that there was a statistically significant difference between smokers and non-smokers in reporting stress as a barrier for quitting smoking at the 6 month time interval (67% *vs.* 46%, p = 0.02), whereas there was no significant difference at 3 and 12 months (78% *vs.* 80%, p = 0.73 and 80% *vs.* 69%, p = 0.19 respectively) [42].

## 4. Discussion

The aim of this systematic review was to bring together the existing body of knowledge regarding the potential association between nursing environment, job stress and nurses’ tobacco use. Workplace, as a stress factor, was examined either as a motivator for starting or increasing smoking habits among nursing staff, or as a barrier for cessation tobacco use. The review of the literature revealed various variables that describe what it is called “job stress”. These variables include heavy physical job strain, long working hours, perceived busyness, perceived work tempo/pressure, poor social climate, shift working, psychological demand, decision latitude, supervisor support, coworker support and patient care activities. That fact could possibly explain the existence of the conflict regarding the potential relationship of stress caused by the workplace and the smoking habits of the nursing staff. Because of the various descriptions of what it is called “job stress” it may be not clear a possible relationship between stress caused by hospital environment and nurses’ tobacco habits.

Studies dealing with the work stress and the smoking behavior of nurses have revealed a link between them. Ota *et al.* found an association between perceived job demand and self-reported nicotine dependence. Nurses with increased levels of high psychological demand may crave tobacco, smoke and became psychologically more dependent on smoking in order to diminish symptoms of mental stress caused by the workplace. Moreover, the relationship between job stress and tobacco use could be stronger in hospital with high smoking prevalence among nurses and among shift workers. Tobacco use may be a way of stress management instead of using other measures of stress reduction [34]. The results of Sarna’s *et al.* study indicated that smoking was not just a personal issue, but it was affecting nurses’ ability to cope with stress. Smoking was described as a way of taking out and coping with the stressful environment of the busy hospital. Cigarettes were described as “stress relieving devices” and the workplace as a place for relapse [40]. According to McKenna *et al.* job stress may not be the causative factor for most nurses’ taking up smoking, but it may play a role in the maintenance of the habit. It seems that job stress had little effect in the etiology of smoking behavior among nurses [20]. Although a strong positive correlation between anxiety scores and smoking that retrieved an anxiolytic influence of smoking was found by the study of Tselebis *et al.*, it can explain only the maintenance of the habit and not the habit of smoking itself [41]. As Steward et al mentioned, smoking has been associated with the relief of anxiety in unselected populations, especially in women [43]. It is well known that smoking is highly addictive and has been marketed as something to promote calm and take a break. Cofta and Staszewski mentioned that hospital staff was smoking more cigarettes in their attempt to find a way of getting away from stress at work. However, there was not a clear way to distinguish the nurses’ involvement, although separate analysis for each category of personnel was performed [38].

Studies that examined the effect of work factors on nurses’ smoking cessation supported a potential link between workplace and tobacco consumption. Eriksen’s surveys indicated that the frequent exposures to threats and violence at work, the lack of supportive, trustful and relaxed social climate in the work unit and the long working hours were associated with increased risk of smoking relapse [36]. Increased risk of experiencing negative emotions could explain an increased risk of relapse [37]. Moreover, long working hours may evoke emotional distress, a well-known inhibitor of smoking cessation [44]. Sanderson *et al.* found that nurses who had heavy physical job strain, as well as those who perceived themselves as having little or no influence on their work were more likely to continue smoking. Two additional job stress related variables: perceived busyness and perceived work tempo/pressure were found to be no statistically significant [39]. Sarna *et al.* concluded that barriers and facilitators to quitting smoking may be present in the workplace, however stress found to be a statistically significant barrier only at 6 month time interval of “quitting smoking using the internet” program by nurses [42].

There are a number of limitations that should be considered in evaluating the review. We used only the computer database of the US National Library of Medicine Medline. We retrieved 491 studies by using the keywords all together and in pairs. By not using a single search string to capture all relevant articles, it may be rather difficult for others to replicate the search. Although language restrictions are generally not recommended in systematic reviews and that is an important limitation, the limits of the review included search on human subjects and English language. Other limitations include the reliance on cross-sectional data for many studies. We also included the survey of Cofta & Staszewski although there was not a clear way to distinguish the nurses’ smoking habit and the possible relationship with stress at hospital, besides of the separate analysis for each category of personnel.

## 5. Conclusions

The systematic review of the literature revealed evidence that the working environment for nurses can be a barrier for cessation and potentially a barrier to maintenance of a previous quit attempt. However, there is a conflict on the possible relationship between workplace as a stressor factor and nurses’ smoking habits, because there is no evidence on if the nurses’ work environment causes smoking initiation. Many nurses and nursing students started smoking before entering hospitals and nursing faculties. Creating a smoke-free and healthy environment will obviously help nurses quit smoking as well as prevent relapse of tobacco use. The recommended methods include use of counseling and support by telephone, internet, other forms of counseling and health provider support and use of pharmacotherapy (nicotine gum, nicotine lozenge, nicotine patch, nicotine inhaler, nicotine nasal spray buproprion). More restricted polices regarding healthcare systems and facilities that promote smoke-free living among nursing staff are necessary to achieve 100% tobacco-free hospitals. It is of great importance to perform further studies for evaluating the time of commencing tobacco consumption by nurses as well as the possible increase of smoking rates among nurses after entering the profession. Such well designed epidemiological studies will obviously help to identify a potential correlation between nurses’ smoking behavior and stress levels in the workplace.

## Figures and Tables

**Table 1.** Summary of characteristics of studies included in the systematic review.

**Authors**	**Main study characteristics**	**Study population**	**Sampling method Hospitals/Departments**	**Smoking status**
Sarna *et al.* (2009)	United States 2004–2006 Prospective cohort study	246 RNs, LPNs, student nurses	Voluntary participation Electronic questionnaire Acute care unit 46% Outpatient 7% Psychiatric unit 8% Other 39%	Smokers (3 months) 58% (6 months) 55% (12 months) 48%
Cofta & Staszewski (2008)	Poland Cross-sectional study	629 employees	Amount-fate method Questionnaire given University of Medical Science Hospital of Lord’s Transfiguration in Poznan	27% smokers 73% nonsmokers
Eriksen (2006)	Norway 1999–2001 Prospective cohort study	1,203 nurses’ aides	Random draw Mailed questionnaire No data available	1,203 nurses’ aides (former smokers)
Sarna *et al.* (2005)	United States 2002 Qualitative study	60 nurses	Voluntary participation Telephone call questionnaire Adult ward 75.5% Pediatric/both 24.5%	50,8% smokers 49,2% ex-smokers
Eriksen (2005)	Norway 1999–2001 Prospective cohort study	2,275 nurses’ aides	Random draw Mailed questionnaire Somatic hospital department (241) Psychiatric hospital (193) Pediatric department (27) Nursing home (966) Old people’s home (232) Community nursing (332) Institution for mentally handicapped (252) Other (127)	90,0% smokers 10,0% ex-smokers
Sanderson *et al.* (2005)	Denmark 1993–1999 Prospective cohort study	4,713 nurses	Danish Nurse Cohort Study Mailed questionnaire No data available about hospitals	24% quit smoking
				11,7% smokers
Ota *et al.* (2004)	Japan 2001 Cross-sectional study	332 female nurses	Participation all of the nurses Anonymous questionnaire given Private General Hospital	88,3% nonsmokers
McKenna *et al.* (2003)	Northern Ireland Cross-sectional study	1,074 nurses	Stratified sample draw Mailed questionnaire Acute hospitals Community trusts Private nursing homes Voluntary sector organizations	25,8% smokers 55,2% nonsmokers 19,0% ex-smokers
McKenna *et al.* (2001)	Northern Ireland Qualitative & quantitative	1,074 nurses	Stratified random sample Distributed questionnaire by means of a liaison person Mailed answers Acute Hospital Sector (72.2%) Community Sector (23%) Private & Voluntary Sectors (3%) N/A (1.9%)	25,8% smokers 19,0% ex-smokers 55,2% nonsmokers
Tselebis *et al.* (2001)	Greece Cross-sectional study	114 female nurses	Random sample State-Trait Anxiety Inventory Scale given Respiratory Diseases Hospital	46,0% smokers 28,0% ex-smokers 26,0% nonsmokers

**Table 2.** Main findings of studies included in the systematic review.

**Study**	**Study topic**	**Statistics**	**Conclusions**
Sarna *et al.* (2009)	Evaluate quit rates at 3, 6 and 12 months following the use of an evidence-based Internet smoking cessation program, describe differences in the use of quit strategies (pharmacotherapy, counseling/skills training, other), including Nurses QuitNet, by smoking status at each time point and identify perceived workplace barriers and facilitators to quitting	a) x^2^ test b) t-test c) Multiple logistic regression analysis	The use of Nurses QuitNet demonstrated promise in supporting quit attempts. Quitting was influenced by workplace factors
Cofta & Staszewski (2008)	Analyze the smoking behaviors of the medical staff in hospitals	a) x^2^ test b) analysis of variance (ANOVA) c) Kruskal-Wallis test	Hospital employees who work on call or at night smoke more cigarettes as a coping mechanism against work stress
Eriksen (2006)	Identify work factors that predict smoking relapse in nurses’ aides	a) x^2^ test b) Multiple logistic regression analysis	A poor social climate in the work unit and frequent exposure to threats and violence at work may be predictors of smoking relapse in nurses’ aides
Sarna *et al.* (2005)	Describe attitudes of nurses about smoking in the workplace	a) x^2^ test b) t-test	Job stress was perceived as diminishing quit attempts and triggering relapse to smoking
Eriksen (2005)	Identify work factors that predict smoking cessation among nurses’ aides	a) x^2^ test b) Fischer’s exact test c) Multiple logistic regression analysis	A negative association between hours of work per week and the odds of smoking cessation in nurses’ aides
Sanderson *et al.* (2005)	Contribute to a better understanding of the significance of lifestyle, health status and work environment on smoking cessation	Multivariate logistic regression analysis	Nurses who perceived themselves as having some or much influence on their work were more likely to quit smoking than those who perceived themselves as having little or no influence
Ota *et al.* (2004)	Examine the relationship between perceived job stress and nicotine dependence by nurses who smoke tobacco	a) Spearman’s correlation coefficients b) Multiple linear regression analysis	Among nurses who smoke tobacco, there was an association between the perceived psychological levels of job demands and the psychological aspects of nicotine dependence
McKenna *et al.* (2003)	Examine the role that peer influence, stress and education levels play in smoking prevalence among nurses	Prevalence rates	Work pressure scored high as a reason for continuing to smoke as did dealing with anxiety/depression. The role of peer pressure, education or stress in the etiology of smoking was not disproved
McKenna *et al.* (2001)	Explore tobacco use and the reasons of smoking by qualified nurses	Means±SD	Work pressure was the third reason for smoking among nurses
Tselebis *et al.* (2001)	Assess the proportion of smokers in nursing staff, assess differences in anxiety levels of nursing staff who have never smoked and assess correlations between anxiety and the number of cigarettes smoked per day in the latter group of nursing staff	a) analysis of variance (ANOVA) b) Multiple linear regression analysis	Nursing staff who were well-acquainted with the ill effects of nicotine abuse, smoking habits was persisting and were correlated with levels of anxiety
